# Formulation and Evaluation of Pharmaceutically Equivalent Parenteral Depot Suspension of Methyl Prednisolone Acetate

**DOI:** 10.4103/0250-474X.51949

**Published:** 2009

**Authors:** A. Alam, Alka Ahuja, Sanjula Baboota, S. K. Gidwani, J. Ali

**Affiliations:** Department of Pharmaceutics, Faculty of Pharmacy, Jamia Hamdard, Hamdard Nagar, New Delhi - 110 062, India; 1 Lupin Research Park, Lupin Ltd., Pune - 411 042, India

**Keywords:** Injectable suspension, methyl prednisolone, DepoMedrol^®^

## Abstract

The aim of the present study was to formulate and evaluate pharmaceutically equivalent injectable aqueous suspension for parenteral depot of methyl prednisolone acetate. Various aqueous suspensions were prepared by rapid stirring and colloid milling method. The prepared aqueous suspensions were subjected to particle size determination, sedimentation study, *in vitro* release studies (pH dependent dissolution study), and stability studies. The optimized formulation consisted of 4% w/w of methyl prednisolone acetate, 2.91% w/w of PEG-3350, 0.19% w/v of injection grade Tween-80, 0.68% w/w of monobasic sodium phosphate, 0.15% w/w of di-basic sodium phosphate, 0.91% w/v of benzyl alcohol, 0.32% w/w sodium meta bisulphate. The *f_2_* value was calculated for innovator (DepoMedrol^^®^^, Batch No. MPH-0254) and optimized formulation at pH 6.8 and pH 7.4 phosphate buffers. The *f_2_* values of 62.94 and 54.37 were obtained at pH 6.8 and pH 7.4 phosphate buffers respectively. The particle size ranged 23-27 μm at D value of 0.9 for both test and innovator product.

Advances have been made in the area of oral controlled release drug delivery systems. However, there are number of possible loopholes in this area of research which includes difficulty in establishing a relationship between *in vivo* and *in vitro* data, unpredictable performance of oral controlled release systems under different dietary conditions, thereby rendering accurate pharmacokinetic prediction and often unpredictable absorption characteristics in different regions of the GIT. Due to these problems in this area, parenteral controlled release systems have been investigated. Methyl prednisolone acetate (MPA), a steroidal anti-inflammatory drug is widely used in musculoskeletal disorders such as arthritis and dysmenorrhea for symptomatic relief of pain and inflammation[1]. Injectable suspensions are heterogonous system, containing solid dispersed phase. They are limited to either subcutaneous or intramuscular routes of administration. Intravenous administration may result in vasoocclusion.[2] Here, for parenteral routes, acetate salt of methyl prednisolone is used. The major drawback of the use of this drug orally is that it undergoes extensive hepatic first pass metabolism and thus only about 50% of the administered dose reaches systemic circulation. In order to avoid this degradation alternative routes have been used and amongst them parenteral route promises significant advantages over the oral route[3]. The parenteral routes are preferred when a rapid and predictable drug response is desired as in an emergency situation, when patient is uncooperative, unconscious, or unable to take drug via an enteral route and when localized drug therapy is required. MPA is a good carrier for depot (Long time action) delivery as it has long biological half life (approx. 46-990 h). The t_1/2_ of MPA was 69.3 hrs, which undergoes substantial hepatic first-pass metabolism, is poorly bioavailable (50-60%), has low molecular weight (416.51) and C_max_ 10-28.5 ng/ml. Injectable, aqueous suspension of MPA have been prepared and studied extensively and it has been concluded that prednisolone can be administered successfully through the parenteral route[4]. Therefore, the aim of present study was to compare particle size, *in vitro* release studies and *f_2_* value calculation of prepared parenteral depot suspension formulation with the innovator product, DepoMedrol^®^ (Batch No. MPH-0254) having varied amount of polymers along with other additives in order to ensure safety and stability of developed formulation. The purpose was to provide the delivery of drug at a controlled rate by intramuscular or subcutaneous route to achieve a therapeutically effective drug level for a longer period of time by injectable suspensions.

## MATERIALS AND METHODS

MPA (Batch No. MPH-0254) was obtained from Lupin Ltd., Pune (India) as a gift sample. Polyethylene Glycol-3350 (PEG-3350)[5,6] was purchased from Sigma Aldrich, USA. Tween-80 was purchased from S. D. Fine Chemicals, Mumbai, India. Monobasic sodium phosphate, dibasic sodium phosphate, benzyl alcohol and sodium metabisulphite were purchased from Qualigens Chemicals, Mumbai, India. All other chemicals and reagents used were of analytical reagent (AR) grade.

### Solubility studies

The solubility of MPA was determined by adding an excess amount of drug in phosphate buffer pH 6.8 and pH 7.4. The flasks were kept on a water bath shaker for 72 h at 37°. After 72 h, solutions were filtered through 0.45 μm membrane filter and aliquots were suitably diluted for estimation of MPA spectrophotometrically at 244 nm.

### Drug excipient interaction studies

The drug excipient interaction[7] studies were performed using thin layer chromatography (TLC) and UV spectroscopy. Mixtures of the drug with different polymers were kept at room temperature, refrigerator temperature, incubator (37°) and oven (50°). The pre-coated HPTLC silica plates were used in the size of 20×20 cm. The pore size of plates was 60 nm. The mobile phase of toulene:ethylacetate:formic acid in the ratio of 50:30:20 was used. After a period of one month, the mixtures were withdrawn and evaluated for appearance, color, odour, gas formation and degradation. The drug excipient mixtures were analyzed by HPTLC method[8].

For the analysis of methyl prednisolone acetate by HPTLC, pre- coated silica gel 60 F (254) plates (E. Merck India, Ltd.) were selected Formic acid, ethyl acetate, toluene, methanol, acetonitrile, diethyl ether were used separately as neat solvents. Developed chromatograms were then visualized in iodine chamber for the detection of methyl prednisolone acetate spots. The R_f_ values were calculated for each chromatogram respectively. Toluene was selected as one of the components of mobile phase as acceptable resolution was obtained. As the R_f_ value was low, the solvent strength was increased by adding polar solvent. Formic acid, ethyl acetate, was added to the toluene in the ratio of 40.0:20.0:20.0, 50.0:40.0:10.0 and 50.0:30.0:20.0 and chromatograms were developed. Good resolution and medium R_f_ range was achieved with the ratio of 50.0:30.0:20.0 (toluene:ethyl acetate:formic acid). Hence this ratio was further chosen as mobile phase for the analysis of methyl prednisolone acetate. Camag Linomat V (Switzerland) sample applicator syringe was used. The volumes between 1-6 μl were applied. The space between the bands was kept at 5 mm. A constant application rate of 150 nl/s was employed. The number of bands applied depended upon the size of plate and on number of tracks required for a particular analysis.

### Preparation of aqueous suspension

Aqueous suspension of MPA containing 40 mg/ml MPA was prepared by dissolving accurately weighed quantity of PEG-3350, Tween 80, monobasic/di-basic sodium phosphate, benzyl alcohol and EDTA in Milli-Q water by continuous stirring. The drug was added during stirring condition in rapid stirrer, at least for half an hour. The formula for MPA suspension is given in Table 1.

**TABLE 1:** DESCRIPTION OF VARIOUS FORMULATIONS PREPARED

Ingredients	Formulations			
	
mg/100 ml	F1	F2	F3	F4
*MPA	4.00	4.00	4.00	4.00
PEG3350	2.91	2.84	3.1	2.91
Polysorbate 80	0.19	0.21	0.19	0.19
Mono-basic-Na-phosphate	0.68	0.62	0.42	0.68
Di-basic-Na-phosphate	0.11	0.14	0.14	0.15
Benzyl alcohol	0.91	0.59	0.91	0.91
Sodium meta bisulphite	0.32	0.32	0.32	0.32

* MPA= methyl prednisolone acetate, PEG= polyethylene glycol, water for injection was used to produce quantity sufficient to 100 ml

### Particle size determination

Various samples like active pharmaceutical ingredient (API), prepared formulation and the innovator product (DepoMedrol^®^ Pharmacia, Batch No. MH-0254) were suspended in Milli-Q water and sonicated to form a smooth and uniform dispersion. The sample was added till obscuration range was within 10-20%. The particle size of the formulation was obtained using a Malvern particle size analyzer (Mastersizer-2000, UK). The pump speed was 2600 rpm. The particle size distributions of the various products were compared at D value of 0.9.

### *In vitro* release studies

**Fig. 1:**
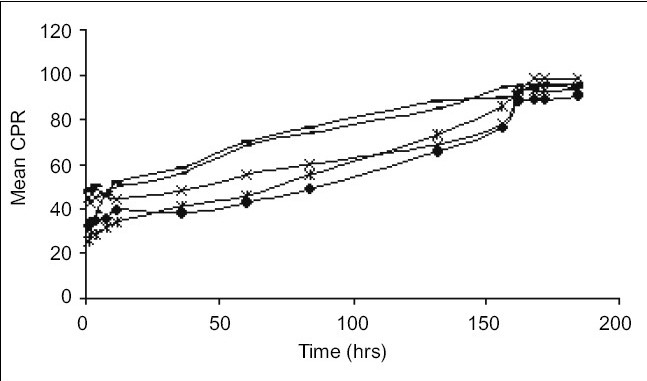
Comparative *in vitro* drug release at pH 6.8. Drug release patterns of F-1 (–×–); F-2(–▪–); F-3 (–=–); F-4 (–◆–); and innovator product (–*–) in phosphate buffer pH 6.8.

**Fig. 2:**
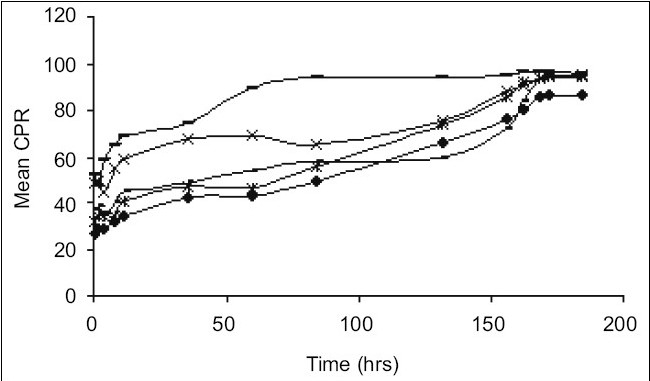
Comparative *in vitro* drug release at pH 7.4. Drug release patterns of F-1 (–×–); F-2(–-–); F-3 (–=–); F-4 (–◆–); and innovator product (–*–) in phosphate buffer pH 7.4.

Release of MPA from various aqueous suspension formulations were studied using dissolution apparatus USP II. Two millilitres of suspension (containing MPA 40 mg/ml) was taken in the flask having 900 ml of phosphate buffer pH 6.8 (fig. 1) and in a separate flask having 900 ml of phosphate buffer pH 7.4 (fig. 2) as medium. The dissolution profile was carried out in two different media in order to simulate muscle and physiological pH. A RPM 50 was adjusted and temperature was maintained at 37±2° throughout the study. Since the dose is very less therefore the quantity of sample taken was 20 ml of aliquots which were withdrawn at predetermined time intervals for a period of 8 days and each time an equal volume was replaced with fresh buffer. The drug content was estimated using a HPLC at wavelength of 254 nm. The chromatographic column used was INT-200 (S. No.W4119 J58 Waters) Symmetry C_8_, particle size 5 μm. The mobile phase was (water:acetonitrile) in the ratio of 60:40. The flow rate was 1.0 ml/min. The retention time was found to be 25 min. The cumulative percent drug releases (CPR) were plotted against time for test and innovator (DepoMedrol^®^) formulations.

### Determination of *f_2_* value for innovator and optimized formulation

The *f_2_* value for innovator product (DepoMedrol^®^) and optimized formulation was determined at pH 6.8 and 7.5. The following formula was used for calculation of *f_2_* value:[9] *f_2_*=50 log[(1+1/nΣ(n)W_t_(R_t_–T_t_)^2^)^−0.5^×100] t=1, where *f_2_* is similarity factor, W_*t*_ is the optional weight factor, n is sum of time points (n=8), R_t_ is percent drug release of reference product and T_t_ is the percent drug release of test product.

### Sedimentation study of optimized formulation

In sedimentation study, the suspension was transferred to a stoppered measuring cylinder and was stored at room temperature (27±1°) for 72 h. The volume of sediment formed was noted at regular interval of time[10]. The sedimentation volume was calculated as the ratio of ultimate height (Hu) of the sediment to the final height (Ho) of the suspension.

## RESULTS AND DISCUSSION

The acetate form of methyl prednisolone was chosen because of its insolubility[7] in water, as depot (long time) action was required. The mean solubility of MPA in phosphate buffer pH 6.8 and pH 7.4 was found to be 2.23±0.16 mg/ml and 1.84±0.12 mg/ml, respectively. Phosphate buffer (pH 6.8 and 7.4) was chosen as the *in vitro* release media. The muscle tissue[12] pH was simulated by phosphate buffer pH 6.8 and physiological tissue pH was simulated by phosphate buffer pH 7.4. HPTLC was performed to assess any interaction between the drug and the excipients. The data obtained suggested that there was no interaction between the drug and the excipients because the R_f_ values of both the drug and drug excipient mixtures were nearly similar (Table 2) Moreover there was no color change, gas formation or any sign of degradation. In particle size study,[13–15] D-value i.e., how many particles of same size exist in particular volume of that sample. D_0.9_ (90% volume) of drug (API), innovator product (DepoMedrol^®^) and test were found to be 7.14, 23.13 and 27.02 μm respectively (Table 3) *In vitro* release studies[16,17] are important for ensuring the depot action performance and the reproducibility of rate and duration of drug release was carried out in alkaline phosphate buffer pH 6.8 and pH 7.4 at 37±2°. The optimized formulation with Type F4 exhibited better depot action at phosphate buffer pH 6.8 (fig. 1) as compared with phosphate buffer pH 7.4 (fig. 2). From *in vitro* drug release study, it was revealed that formulation exhibited best release when compared with other formulation. Cumulative percent drug release in 184 h (8 days) from innovator (DepoMedrol^®^) and test product were 93.3 and 90.0% at pH 6.8 and 80.2 and 94.2% at pH 7.4 phosphate buffer, respectively. The *f_2_* value is a measurement of the similarity between the dissolution profiles of two true profiles (test and innovator). The *f_2_* value was found to be 62.94 and 54.87 at pH 6.8 (Table 4) and pH 7.4 (Table 5), respectively. The value shows a similarity between the dissolution profiles of optimized formulation (F4) with the innovator (DepoMedrol^®^) product. The sedimentation volume was found to be constant from 0.72 to 0.50 for a period of 72 h (Table 6). Stability studies of optimized formulation revealed that no significant changes occured in physiochemical properties like crystal growth, sedimentation, particle size (20±10 μm) and pH (6.25±1.0) of the optimized formulation at various storage temperatures for a period of three months (Table 7).

**TABLE 2:** DRUG EXCIPIENT INTERACTION BY HPTLC METHOD

Temperature	R_f_ values for different formulations			
	
	F1	F2	F3	F4
Refrigerator	0.915	0.898	0.854	0.798
Room	0.886	0.895	0.873	0.796
40/75%	0.892	0.902	0.815	0.764
30/65%	0.854	0.887	0.912	0.782
25/60%	0.823	0.794	0.853	0.669

**TABLE 3:** PARTICLE SIZE ANALYSIS OF PURE DRUG, INNOVATOR PRODUCT AND FORMULATIONS

Sample	Source	Bulk lot	Obscuration	D _(0.9)_ ±SD
*MPA (API)	Pure drug	BK-031	18.74	07.141 ± 1.1
**Depo-Medrol	Pharmacia	LK-099	13.58	22.986 ± 2.3
**Depo Medrol	Pharmacia	LK-034	13.06	23.131 ± 3.5
Formulation-F1	In-house	AFT-F1	13.26	38.067 ± 6.2
Formulation-F3	In-house	AFT-F2	16.51	62.524 ±7.9
Formulation-F3	In-house	AFT-F3	13.83	53.675 ± 7.6
Formulation-F4	In-house	AFT-F4	13.68	27.021 ± 9.8

* MPA (API)= methyl prednisolone acetate (active pharmaceutical ingredient)

** Depo Medrol^®^= Innovator product

**TABLE 4:** *F_2_* VALUE CALCULATION AT PH 6.8 PHOSPHATE BUFFER (MEAN CPR)*

Time(t)	*f_2_* value of optimized formulation (F4)								
	
	n 1	n 2	n 3	n 4	n 5	n 6	n 7	n 8	Sum
Reference	26.9	28.8	29.2	32.1	34.5	42.7	66.5	88.4	-
Test	31.9	34.6	34.9	36.1	40.1	46.8	46.8	73.9	-
Rt-Tt	−5.0	−5.7	−5.7	−4.0	−5.6	−4.1	−7.4	−5.1	-
(Rt-Tt)^2^	25.0	32.49	32.49	16.0	31.36	16.81	54.76	26.01	234.92

*f_2_*=50*log{[1+(1/nsum(Rt-Tt)^2]−0.5}*100=62.94066786

* CPR= Cumulative percent release, n1-n8= Time points, n= Mean time points

**TABLE 5:** *F_2_* VALUE CALCULATION AT PH 7.4 PHOSPHATE BUFFER (MEAN CPR)*

Time(t)	*f_2_* value of optimized formulation (F4)								
	
	n 1	n 2	n 3	n 4	n 5	n 6	n 7	n 8	Sum
Reference	23	33.4	43.9	47.7	68.9	82.7	86.1	96.6	-
Test	28.1	33.9	36.4	49.6	50.6	73.7	87.6	97.0	-
Rt-Tt	−5.1	−5.1	7.5	−1.9	18.3	9.0	−1.5	−0.4	-
(Rt-Tt)^2^	26.01	0.25	56.25	3.61	334.89	81.0	22.5	0.16	234.92

*f_2_*=50*log{[1+(1/nsum(Rt-Tt)^2]-0.5}*100

* CPR= Cumulative percent release, n1-n8= Time points, n= Mean time points

**TABLE 6:** SEDIMENTATION STUDY ANALYSIS

Time (h)	Sedimentation volume (Hu/Ho)
0.5	0.72
12	0.70
24	0.68
30	0.60
48	0.59
54	0.50
72	0.50

**TABLE 7:** STABILITY STUDIES OF OPTIMIZED FORMULATION (F4)

Time point	Storage (Temp/RH)	Crystal Formation	pH	Particle size (μm)	Sedimentation
0 day	-	Nil	6.34	23.13	Nil
30 days	40°/75%	Nil	6.26	22.56	Nil
	30°/65%	Nil	6.31	23.48	Nil
	25°/60%	Nil	6.20	21.63	Nil
	5°	Nil	6.19	20.31	Nil
60 days	40°/75%	Nil	6.14	24.95	Nil
	30°/65%	Nil	6.25	23.14	Nil
	25°/60%	Nil	6.19	26.53	Nil
	5°	Nil	6.13	21.37	Nil
90 days	40°/75%	Nil	6.08	25.48	Nil
	30°/65%	Nil	6.12	26.31	Nil
	25°/60%	Nil	6.21	22.14	Nil
	5°	Nil	6.15	23.92	Nil
